# CUBIC pathology: three-dimensional imaging for pathological diagnosis

**DOI:** 10.1038/s41598-017-09117-0

**Published:** 2017-08-24

**Authors:** Satoshi Nojima, Etsuo A. Susaki, Kyotaro Yoshida, Hiroyoshi Takemoto, Naoto Tsujimura, Shohei Iijima, Ko Takachi, Yujiro Nakahara, Shinichiro Tahara, Kenji Ohshima, Masako Kurashige, Yumiko Hori, Naoki Wada, Jun-ichiro Ikeda, Atsushi Kumanogoh, Eiichi Morii, Hiroki R. Ueda

**Affiliations:** 1 Department of Pathology, Graduate School of Medicine, Osaka University, 2-2 Yamadaoka, Suita, Osaka, 565-0871 Japan; 2 Department of Immunopathology, WPI Immunology Frontier Research Center, Osaka University, 3-1 Yamadaoka, Suita, Osaka, 565-0871 Japan; 3 Department of Systems Pharmacology, Graduate School of Medicine, The University of Tokyo, 7-3-1 Hongo, Bunkyo-ku, Tokyo, 113-0033 Japan; 4 Laboratory for Synthetic Biology, RIKEN Quantitative Biology Center, 1-3 Yamadaoka, Suita, Osaka, 565-0871 Japan; 5 PRESTO, Japan Science and Technology Agency (JST), 4-1-8 Honcho, Kawaguchi, Saitama, 332-0012 Japan; 6 Department of Pathology, Kinki Central Hospital, 3-1 Kurumazuka, Itami, Hyogo, 664-8533 Japan; 7 Department of Surgery, Kinki Central Hospital, 3-1 Kurumazuka, Itami, Hyogo, 664-8533 Japan; 8 Department of Nutrition Oncology, Osaka International Cancer Institute, 3-1-69, Otemae, Chuo-ku, Osaka-shi, Osaka, 541-8567 Japan; 9 Department of Respiratory Medicine, Allergy and Rheumatic Disease, Graduate School of Medicine, Osaka University, 2-2 Yamadaoka, Suita, Osaka, 565-0871 Japan

## Abstract

The examination of hematoxylin and eosin (H&E)-stained tissues on glass slides by conventional light microscopy is the foundation for histopathological diagnosis. However, this conventional method has some limitations in x-y axes due to its relatively narrow range of observation area and in z-axis due to its two-dimensionality. In this study, we applied a CUBIC pipeline, which is the most powerful tissue-clearing and three-dimensional (3D)-imaging technique, to clinical pathology. CUBIC was applicable to 3D imaging of both normal and abnormal patient-derived, human lung and lymph node tissues. Notably, the combination of deparaffinization and CUBIC enabled 3D imaging of specimens derived from paraffin-embedded tissue blocks, allowing quantitative evaluation of nuclear and structural atypia of an archival malignant lymphoma tissue. Furthermore, to examine whether CUBIC can be applied to practical use in pathological diagnosis, we performed a histopathological screening of a lymph node metastasis based on CUBIC, which successfully improved the sensitivity in detecting minor metastatic carcinoma nodules in lymph nodes. Collectively, our results indicate that CUBIC significantly contributes to retrospective and prospective clinicopathological diagnosis, which might lead to the establishment of a novel field of medical science based on 3D histopathology.

## Introduction

Hematoxylin and eosin (H&E) staining, together with other assistive techniques such as immunohistochemistry or *in situ* hybridization, is the foundation for histopathological diagnosis. Pathologists examine stained tissue preparations with bright-field microscopy to estimate various types of histological and pathological findings such as cell malignancy, degree of inflammation and fibrosis, depth of tumor invasion, presence of tumor components in the cut margin, and lymph node metastasis status. Although these conventional methods provide plenty of information about morphologic changes of cells and tissues, they also have fundamental limitations. For instance, the conventional methods can provide only planar 2-dimentional (2D) images, limiting their ability to observe 3-dimentional (3D) structures consisting of a variety of cells in diverse anatomical structures. In addition, particularly in a histopathological diagnosis for large pathological specimens such as surgically resected whole organ specimens, only representative lesions identified by a macroscopic observation are typically evaluated. Therefore, a concern that an additional critical lesion may be present in the non-evaluated areas still remains to be addressed.

To enable more comprehensive and efficient pathological diagnosis, there have been efforts to serially section and image relatively large samples, followed by the reconstruction of 3D images. Early cases of such efforts were performed in the late 1960s and 1970s, in which gross images were matched with corresponding histological sections of 6–8 μm-thickness^1–3^. Today, it is possible to generate reconstructed 3D images by computer-processed imaging of serially sectioned tissue, which enables 3D histopathological observation of lesions, such as breast carcinoma, in biopsy specimens^4–7^. However, this section-based method does not enable perfectly continuous observation of tissues structures due to its inevitable discontinuity caused by tissue sectioning, although it enables the observation of the rough structure of lesions, such as tumor volume, branches of tumor nodule, and presence or absence of lumina inside tumors^7^. Moreover, to obtain such reconstructed 3D images by this method, hundreds of glass slides from serially sectioned tissues are needed, which is a high cost and labor-intensive process.

On the other hand, remarkable advances have been recently made in tissue-clearing techniques^8–24^. These can be classified by (1) hydrophobic reagents, (2) hydrophilic reagents, or (3) hydrogel-based methods, and have different characteristics^8, 25^. These techniques make an organ transparent so that light can illuminate cells deep in the tissues. Therefore, it enables the acquisition of volumetric images with various optical microscopies such as confocal fluorescence microscopy, multiphoton fluorescence microscopy, and light-sheet fluorescence microscopy (LSFM).

The advantages of such tissue-clearing and 3D-imaging techniques have been probed in a several pathological studies. For example, the change of islets’ volume and number in a 3D image of a whole pancreas from streptozotocin-induced diabetic mouse has been quantitatively evaluated^9^. 3D imaging also enabled quantification of abnormal ladder-shaped structures formed by neurites in a human autism patient^14^. More recently, quantitative analyses of β-amyloid plaque distribution, structure, heterogeneity, and spatial relationship with glial cells and vessels were performed in the brains of a mouse model and human patients of Alzheimer’s disease^11, 16^. Other groups also demonstrated the advantages of 3D observation with normal and patient-derived tissues^16, 26–31^. For more practical use in clinical pathology, compatibility with paraffin-embedding or H&E staining were also tested^27, 32, 33^. Despite these potential applications, the emerging tissue-clearing and 3D-imaging techniques have been applied to only a small number of pathological samples, and there are no reports demonstrating that the techniques practically contribute to the improvement of sensitivity and/or specificity of clinicopathological examination, particularly in a comprehensive study using a large number of pathological samples.

To validate the applicability of these techniques to 3D clinical diagnostic pathology, we applied the Clear, Unobstructed Brain/Body Imaging Cocktails and Computational analysis (CUBIC) pipeline to human organs and evaluated its efficacy on pathological specimens. CUBIC is a hydrophilic tissue-clearing and 3D-imaging technique, which offers rapid and high-performance whole-organ and whole-body 3D imaging^9, 34^. Its usefulness in deep and comprehensive imaging of murine organs including brain, heart, and liver has been already evaluated by several groups^35–37^. In fact, we demonstrate, in this study, that the current version of CUBIC protocol efficiently cleared human lung and lymph node tissues, whose detailed structures such as bronchi, vasculature, or lymphoid follicles were successfully visualized in 3D. CUBIC was also applicable to specimens derived from paraffin-embedded tissue blocks. This method enabled the evaluation of nuclear and structural atypia of a malignant lymphoma tissue prepared from an archival paraffin-embedded tissue block. In addition, the CUBIC-based screening of lymph node metastasis improved the sensitivity for detection of metastatic carcinoma nodules in lymph nodes. Thus, CUBIC significantly contributes to both retrospective and prospective clinicopathological diagnosis. We also note that CUBIC did not impair a routine pathological examination because conventional H&E evaluation was successfully performed even after tissue clearing by CUBIC. These results suggest that tissue-clearing and 3D-imaging techniques can expand the coverage of standard histopathological examination from 2D to 3D as well as from a limited area to a wider region, and hence potentially contribute to more accurate clinical diagnosis.

## Results

### Tissue clearing of various human organs with CUBIC

**Figure 1:**
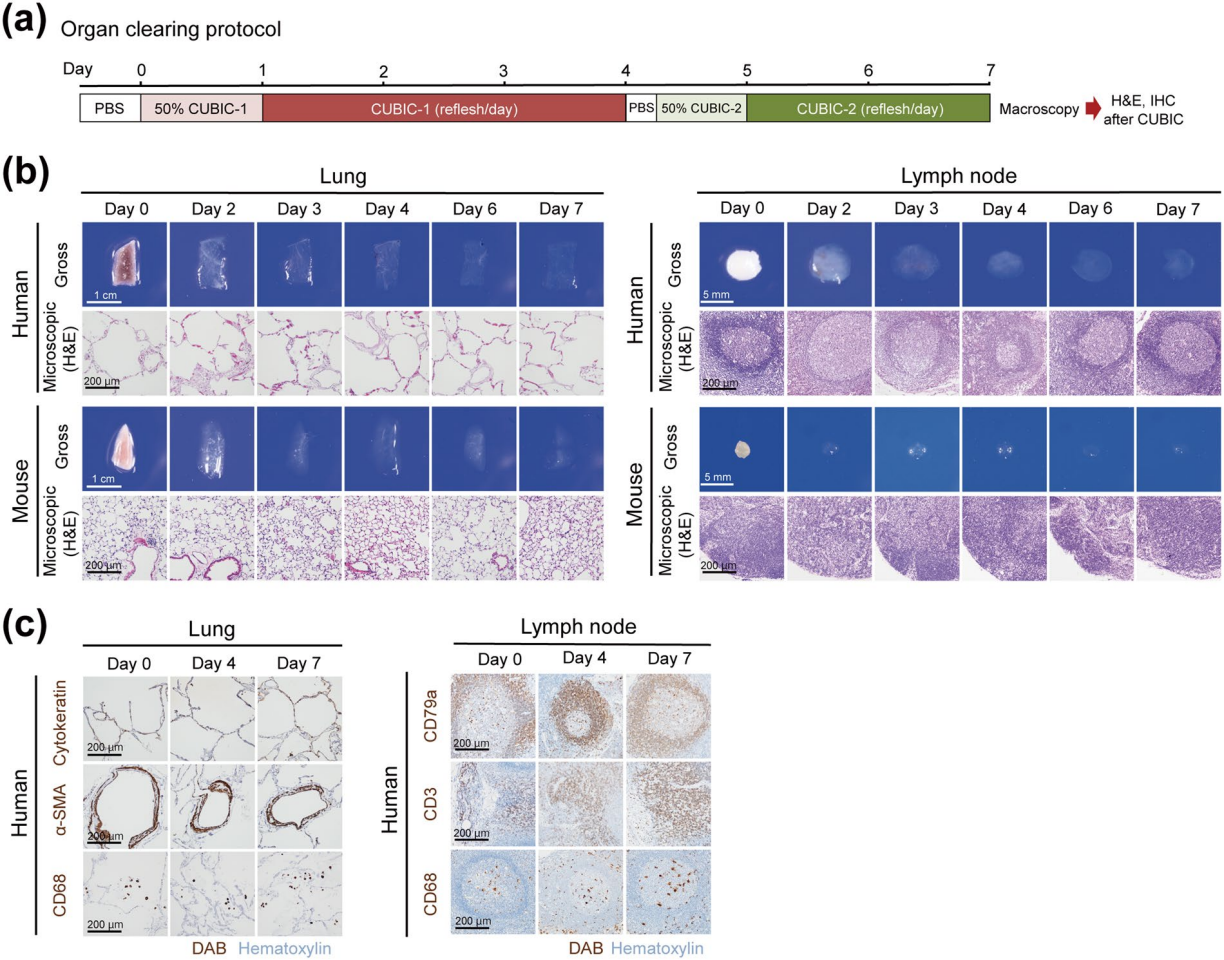
Tissue clearing of various human organs with CUBIC. (**a**) Schematic diagram of clearing protocol for human organs. IHC, immunohistochemistry. (**b**) Gross and microscopic images of human or mouse lung and lymph nodes. After gross image acquisition at the indicated time points, lung and lymph node tissues were washed with PBS, followed by paraffin embedding, sectioning, and H&E staining. (**c**) Representative images of immunohistochemistry of human lung and lymph node tissues after clearing by CUBIC. Serial sections prepared from lung or lymph node tissues which were histologically evaluated in (**b**) were immunostained with anti-cytokeratin (epithelial cell marker), anti-α-SMA (smooth muscle cell marker), or anti-CD68 (monocyte/macrophage marker) antibodies (lungs), or immunostained with anti-CD79a (B-cell marker), anti-CD3 (T-cell marker), or anti-CD68 (monocyte/macrophage marker) antibodies (lymph nodes). DAB, 3,3′-diaminobenzidine.

We first tested the compatibility of the first version of the CUBIC protocol^9, 34, 38^ for various human organs. 2–5 mm-thick tissue blocks cut out from formaldehyde-fixed human brains, hearts, lungs, livers, kidneys, spleens, intestines, and lymph nodes were subjected to tissue-clearing procedure according to the protocol described in the original paper (Fig. 1a)^34^. Among these organs, lung and lymph node tissues were significantly cleared with the efficiency comparable to that of mouse organs (Fig. 1b). Furthermore, these cleared tissues can be subjected to paraffin-embedding, sectioning, and H&E staining without any problems after the release from CUBIC clearing by PBS wash. Images of the H&E-stained sections indicated that the CUBIC clearing caused only negligible, if any, degeneration of the tissues (Fig. 1b and Supplementary Fig. 1), suggesting that application of CUBIC to the pathological specimens does not lead to any disadvantages for the subsequent H&E staining, which is required in routine pathological diagnosis in hospitals. In addition, tissue clearing with CUBIC preserved the antigenicity of some of diagnostic markers such as Cytokeratin, α-SMA, CD68, CD79a and CD3 in these tissues (Fig. 1c), suggesting that pathologists may perform immunohistochemistry for accurate diagnosis even after tissue clearing with CUBIC. These results are consistent with a previous study using CUBIC-cleared murine mammary gland tissues^39^. Taken together, the CUBIC clearing protocol can be applied to some human tissues without any disadvantages for routine procedures of histopathological examination. On the other hand, the first version of CUBIC reagents did not completely clear a human brain, heart, liver, kidney, spleen, and intestine as efficiently as lungs and lymph nodes (Supplementary Fig. 1). Although it was reported that CUBIC reagents enable efficient decolorization of endogenous chromophores (e.g., heme) inside murine tissues^9^, endogenous chromophores in human heart, liver, and spleen were not completely decolorized. The issue in tissue-clearing performance for these tissues will be addressed in future studies to update CUBIC reagents with more efficient clearing capability. In the following experiments of this study, we primarily used lung and lymph node to test the further feasibility of 3D pathology with CUBIC.

### 3D imaging of various human organs with CUBIC

**Figure 2:**
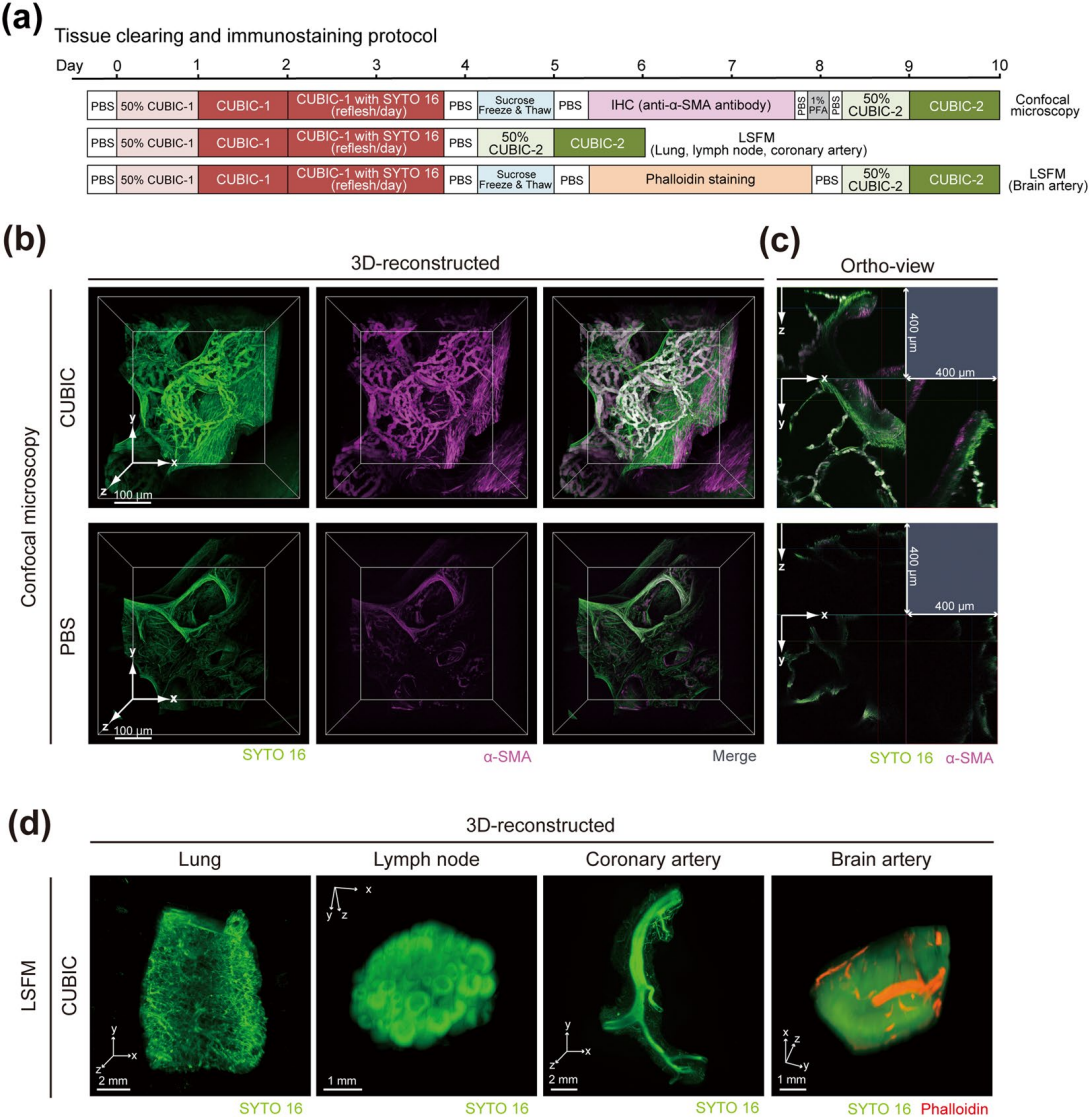
3D imaging of various human organs with CUBIC. (**a**) Schematic diagram of clearing and immunostaining protocol for human lungs, lymph nodes, and peripheral arteries. IHC, immunohistochemistry. (**b**,**c**) The reconstructed 3D images and orthogonal section images of human lung tissue blocks stained with Alexa Fluor 647-conjugated anti-α-SMA antibody and SYTO 16, with or without clearing by CUBIC. Images were obtained by confocal microscopy (z-stack: 1 μm/slice). (**d**) The reconstructed 3D images of SYTO 16-stained human lung tissue block, SYTO 16-stained half-cut human mesenteric lymph node, SYTO 16-stained peripheral branch of coronary artery which was dissected from a human heart, and SYTO 16- and Alexa Fluor 594-labeled phalloidin-stained peripheral arteries in the surface of human cerebral cortex block. Images were obtained by light-sheet fluorescence microscopy (LSFM) (z-stack: 5 μm/slice).

Next, we tested whether the 3D imaging can be performed on cleared normal human tissues. First, we cleared tissue blocks of human lung based on the CUBIC clearing protocol (Fig. 2a **)**. We then performed immunostaining with an anti-α-smooth muscle actin (α-SMA) antibody and counterstaining of cell nuclei with a cell-permeable green-fluorescent nucleic acid stain SYTO 16 (Fig. 2a **)**. We imaged the cleared and stained lung samples by confocal microscopy and LSFM. As shown in Fig. 2b and Supplementary Movie 1, reconstructed 3D images obtained by confocal microscopy successfully visualized detailed 3D tissue structures including the network of bronchial branches, vasculature, and interstitial fibers in the SYTO 16-stained human lung tissue. Immunohistochemistry with an anti-α-SMA antibody allowed selective labeling of the network of blood vessels running through the interstitial spaces (Fig. 2b). We could observe lung tissue structures as deep as at least 400 μm when the tissue was cleared by CUBIC, whereas only scattered fluorescent signals were detected at maximum depth of 150 μm without tissue clearing (Fig. 2b,c). In addition, the tile scanning tool of the confocal microscopy allowed us to image a larger area of the tissue with the same resolution (Supplementary Fig. 2).

We also performed clearing, staining and imaging with LSFM for acquiring an entire 3D image of several human tissues. Reconstructed 3D images of SYTO 16-stained human lung, lymph node and coronary artery of the heart visualized the macroscopic anatomical features of each tissue; networks of alveolus, bronchi, and vasculature in the lung, lymphoid follicles in the lymph node, and peripheral branches of coronary artery (Fig. 2d, Supplementary Movie 2, and Supplementary Movie 3). Furthermore, actin staining by phalloidin helped us to selectively label small arteries in the surface of human brain cortex, which were also visualized in reconstructed 3D images (Fig. 2d). Taken together, CUBIC is applicable to 3D histology and anatomy of various human organs.

### Tissue clearing and 3D imaging of pathological specimens with CUBIC

**Figure 3:**
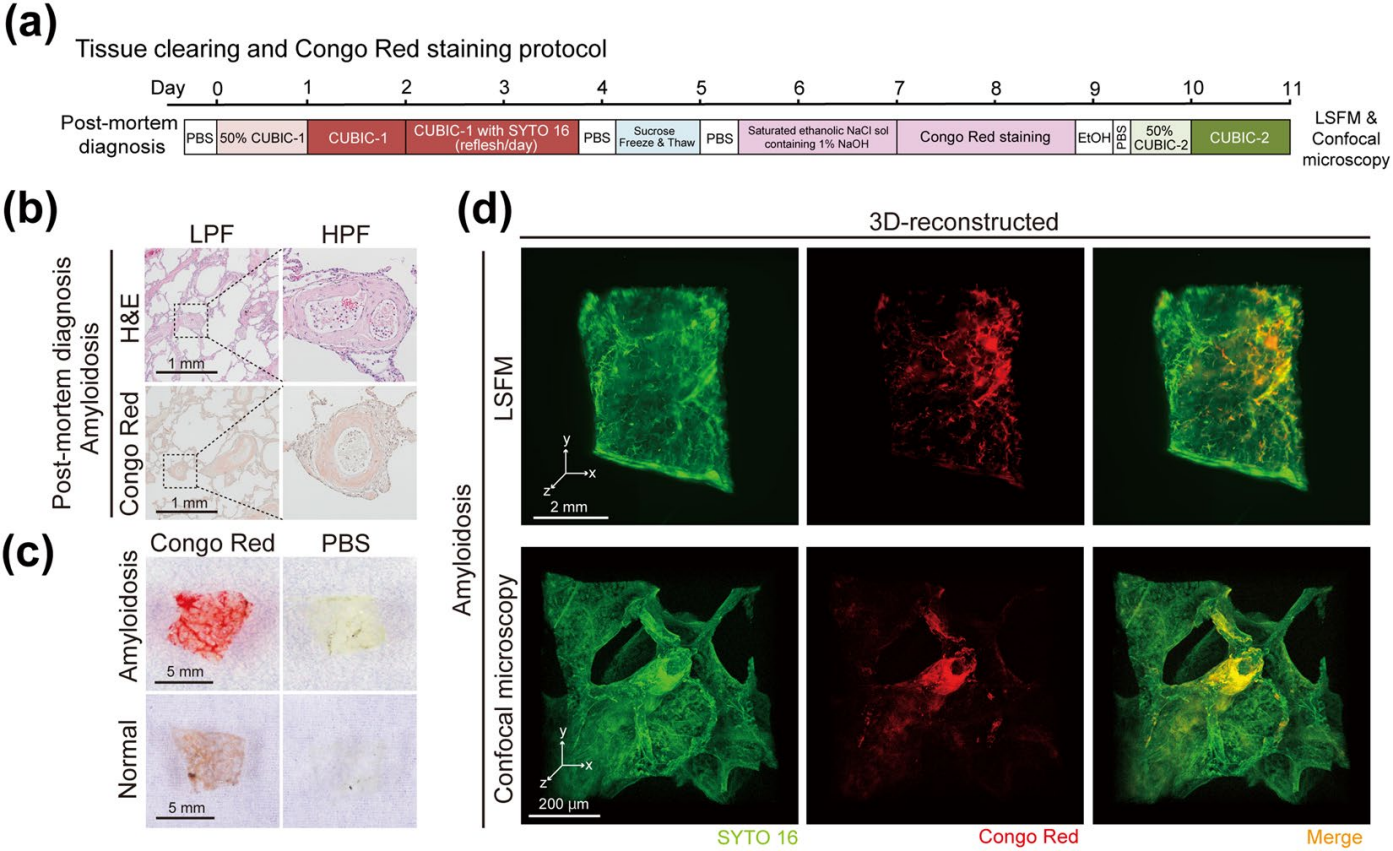
Tissue clearing and 3D imaging of pathological specimens with CUBIC. (**a**) Schematic diagram of clearing and Congo Red staining protocol for human tissue blocks of lung amyloidosis. (**b**) Representative histopathological images of this case of lung amyloidosis, which was obtained from glass slides generated for post-mortem diagnosis. H&E staining (upper) and Congo Red staining (lower) show pink-colored amyloid deposition in blood vessel walls in lung. (**c**) Gross images of amyloidosis patient-derived and normal lung tissue blocks after Congo red staining or PBS soaking. An image of an amyloidosis patient-derived lung tissue block (upper, left) shows that arteries are macroscopically visible in red. (**d**) The reconstructed 3D images of human lung tissue block stained with Congo Red, showing that amyloid deposition in artery wall in lung. The images were obtained by LSFM (z-stack: 5 μm/slice) (upper panels) or confocal microscopy (z-stack: 1 μm/slice) (lower panels).

We next explored the applicability of CUBIC to pathological specimens derived from patients. Since lung and peripheral arteries were efficiently cleared and visualized as shown above, we focused on lung amyloidosis. This disease is characterized by the deposition of an abnormal protein called amyloid in the blood vessel walls and connective tissues, resulting in systemic organ dysfunction. To visualize the amyloid deposits in blood vessel walls, blocks of the patient’s lung tissue remaining after usual pathological diagnosis were subjected to clearing and whole-mount Congo Red staining (Fig. 3a). Congo Red is commonly used for amyloid staining in the usual pathological diagnosis together with H&E staining (Fig. 3b). Congo Red staining worked efficiently even after CUBIC clearing; the amyloid deposits in the tissue block were macroscopically visible in the specimen (Fig. 3c). 3D imaging of the cleared and stained samples using confocal microscopy or LSFM showed tubular amyloid deposition in the blood vessel walls of the lung (Fig. 3d and Supplementary Movie 4). A similar image was also acquired by using Thioflavin T staining, another fluorescent dye used for amyloid staining (Supplementary Fig. 3). These results indicate that CUBIC enabled 3D imaging of pathological specimens derived from patients.

### Tissue clearing and 3D imaging of deparaffinized samples with CUBIC

Another attractive challenge in pathological examination is to expand the application of the tissue-clearing and 3D-imaging techniques to pathological specimens from paraffin-embedded tissue blocks. As routine pathological procedure in hospitals, tissues or organs removed from patients are fixed with formaldehyde, and subjected to macroscopic observation in order to select and trim the representative pathological regions. The selected area of tissues is then subjected to paraffin-embedding, sectioning, and H&E staining. These paraffin-embedded tissue blocks of various diseases are thus archived in the department of pathology in hospitals all over the world. Therefore, we next tested whether CUBIC was applicable to these paraffin-embedded archives toward retrospective and collective examination of 3D histopathology.

**Figure 4:**
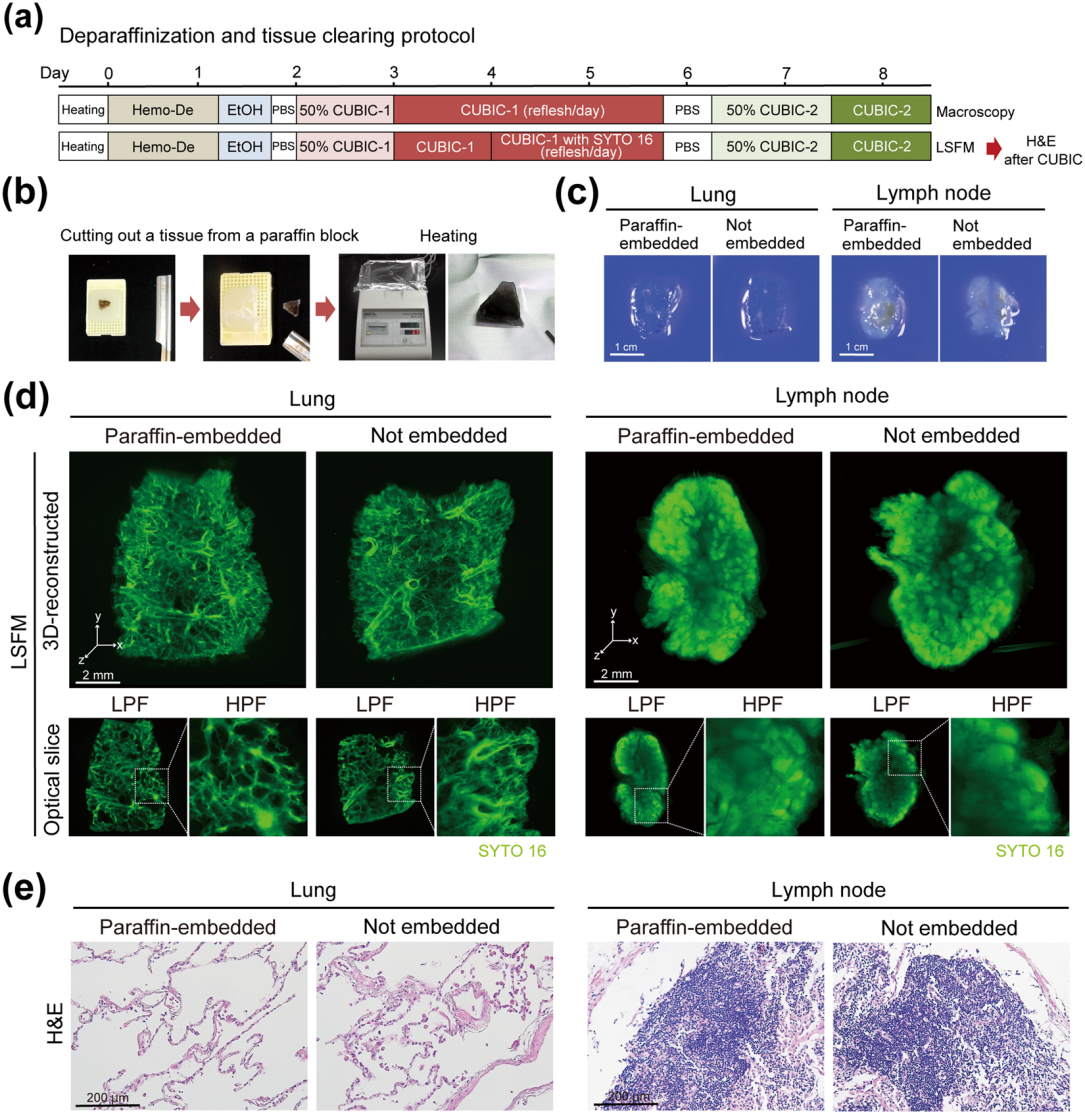
Tissue clearing and 3D imaging of deparaffinized samples with CUBIC. (**a**) Schematic diagram of deparaffinization and subsequent tissue clearing of specimens derived from paraffin-embedded tissue blocks. (**b**) Tissues were cut out from paraffin-embedded tissue blocks with a blade, followed by heating at 65 °C on a heat block until paraffin was macroscopically removed from tissues. (**c**) Gross images of human lung and lymph node tissue blocks after deparaffinization and tissue clearing procedures. Formaldehyde-fixed human lung or lymph node tissues were cut in half. One was embedded into paraffin block, followed by deparaffinization and tissue clearing according to the protocol shown in (**a**). The other was kept in PBS instead of paraffin-embedding, followed by the tissue clearing. (**d**) The reconstructed 3D images of SYTO 16-stained lung tissue blocks with or without paraffin embedding and deparaffinization steps. Formaldehyde-fixed human lung or lymph node tissues were cut in half. One half was subjected to paraffin-embedding and deparaffinization before clearing. Then, the samples were cleared, stained with SYTO 16 and imaged with LSFM (z-stack: 5 μm/slice) according to the protocol shown in (**a**). x-y plane optical slices (Lung, Paraffin-embedded, slice #375, z = 1.875 mm; Lung, Not embedded, slice #275, z = 1.375 mm; Lymph node, Paraffin-embedded, slice #425, z = 2.125 mm; Lymph node, Not embedded, slice #315, z = 1.575 mm) are also presented. (**e**) Representative images of H&E staining of the human lung and lymph node tissues after clearing and imaging in (**d**).

We first prepared tissue blocks from formaldehyde-fixed normal human lung and lymph node. These blocks were then cut in half. One half was kept in PBS whereas another half was first embedded into a paraffin block, was recovered by deparaffinization, and then was subjected to tissue clearing by CUBIC (Fig. 4a,b). The gross appearance of the resulting samples was not distinguishable and had an equivalent transparency (Fig. 4c). Note that a paraffin-embedded and deparaffinised specimen but without CUBIC-1 step was not efficiently cleared, suggesting the necessity of further delipidation by CUBIC-1 despite an expectation that a certain amount of lipids would be removed through paraffin-embedding process (Supplementary Fig. 4). Next, we performed LSFM imaging to obtain 3D images of cleared lung and lymph node stained by SYTO 16, which demonstrated similar image quality (Fig. 4d). We also confirmed that the degree of tissue degeneration in the H&E section after the CUBIC procedure was basically equivalent between the tissues with or without paraffin-embedding and deparaffinization steps (Fig. 4e, Supplementary Fig. 5). These results indicated that paraffin-embedded tissues in pathology archives can be used for CUBIC procedures after a suitable deparaffinization and recovery procedures.

### Quantitative analysis of cellular atypia in an archival paraffin-embedded malignant lymphoma tissue

**Figure 5:**
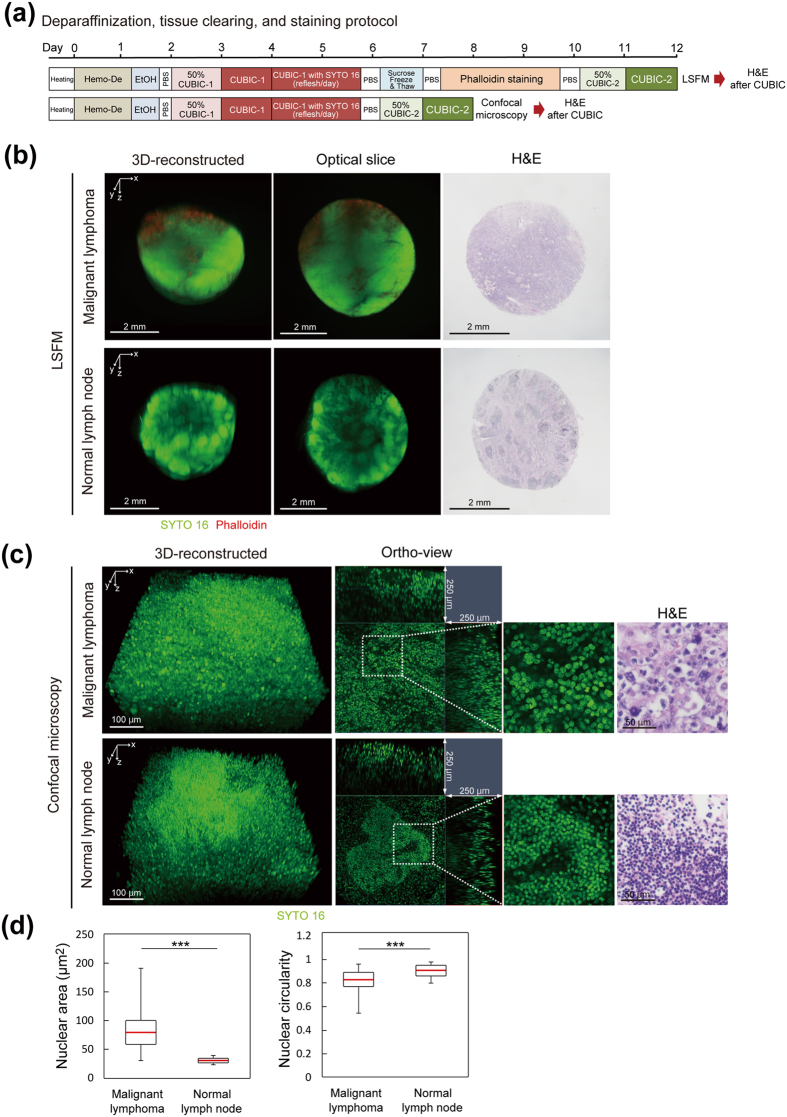
Tissue clearing and 3D imaging of paraffin-embedded tissues in pathology archives with CUBIC. (**a**) Schematic diagram of deparaffinization and subsequent tissue clearing and staining of specimens derived from paraffin-embedded tissue blocks. (**b**) The reconstructed 3D images and representative optically sliced images of malignant lymphoma or control normal lymph node tissues derived from paraffin-embedded tissue blocks, obtained by LSFM imaging (z-stack: 5 μm/slice). The tissues were subjected to deparaffinization, clearing, and staining according to the protocol shown in (**a**). x-y plane optical slices (Malignant lymphoma, slice #250, z = 1.25 mm; Normal lymph node, slice #250, z = 1.25 mm) are also presented. After the imaging, the tissue was washed with PBS and used for the subsequent H&E staining. (**c**) The reconstructed 3D images and orthogonal section images of malignant lymphoma and control normal lymph node tissues derived from paraffin-embedded tissue blocks. Images were obtained by confocal microscopy (z-stack: 1 μm/slice). After the imaging, the tissue was washed with PBS and used for the subsequent H&E staining. (**d**) Quantitative analysis of the area and circularity of malignant lymphoma cells and normal lymphocytes. 10 independent optical slices were selected from the z-stacked image shown in (**b**). 10 cells per respective slices were randomly selected and the areas and circularity of these cells were measured using Image J software. Circularity is defined as the formula: circularity = 4π(area/perimeter^2^), which gives the value = 1 when the object is a precise circle. Means of two sets of data were statistically evaluated with Welch’s t-test, because Kolmogorov-Smirnov test and F-test demonstrated that the two groups are normal distributions without equal variance. ***P < 0.01 (Welch’s t-test).

The successful tissue clearing and 3D imaging of pathological specimens derived from paraffin-embedded tissues led us to test the retrospective histopathological examination of patient-derived tissues recovered from paraffin-embedded tissues stored in the pathological archives of hospitals. We used a paraffin-embedded tissue block of malignant lymphoma stocked for years, together with a newly-prepared normal lymph node block as a control. Malignant lymphoma is a lymph tissue-origin neoplasm and histopathologically characterized by abnormal morphology such as the disordered histological structure (structural atypia), the morphological changes of nucleus (nuclear atypia), and the variability in the size and shape of the malignant cells (pleomorphism). Since lymphoma cells have scant cytoplasm, the degree of nuclear atypia and pleomorphism is particularly important in the diagnosis of malignant lymphoma. To test whether these hallmarks could be observed and quantified by 3D imaging, we recovered, cleared and imaged the paraffin-embedded lymph nodes with CUBIC (Fig. 5a **)**. The tissues were punched out from paraffin-embedded tissue blocks using a biopsy punch with 4 mm diameter in order to minimize the loss of tissues (Supplementary Fig. 6). We obtained reconstructed 3D images and representative 2D optical slice images by LSFM and found that malignant lymphoma cells demonstrated diffusely infiltrating pattern, which reflects their structural atypia, while lymphocytes in the normal lymph node formed lymphoid follicles (Fig. 5b). In addition, phalloidin-stained actin fibers of infiltrating fibroblast cells were regionally visible in a lymphoma specimen, which helped us to evaluate the degree of fibrosis induced by invasion of lymphoma cells (Fig. 5b). Section images of conventional H&E staining after CUBIC gave consistent results with these histopathological features (Fig. 5b). Furthermore, images with higher resolution by confocal microscopy showed more precise nucleic morphology of the cells; lymphoma cells showed nuclear atypia and pleomorphism, as compared with normal lymphocytes with more uniform nucleic shape and size (Fig. 5c). Statistical analysis from the stacked images confirmed the abnormality in the nuclear shapes of lymphoma cells, such as increased nuclear size and decreased nuclear circularity (nuclear atypia), and larger variance of these parameters (nuclear pleomorphism) (Fig. 5d). These findings indicated that 3D pathological examination of clinical specimens stored in the paraffin-embedded archives could be performed with the CUBIC pipeline.

### 3D clinicopathological screening of metastatic carcinoma nodules in lymph nodes with CUBIC

**Figure 6:**
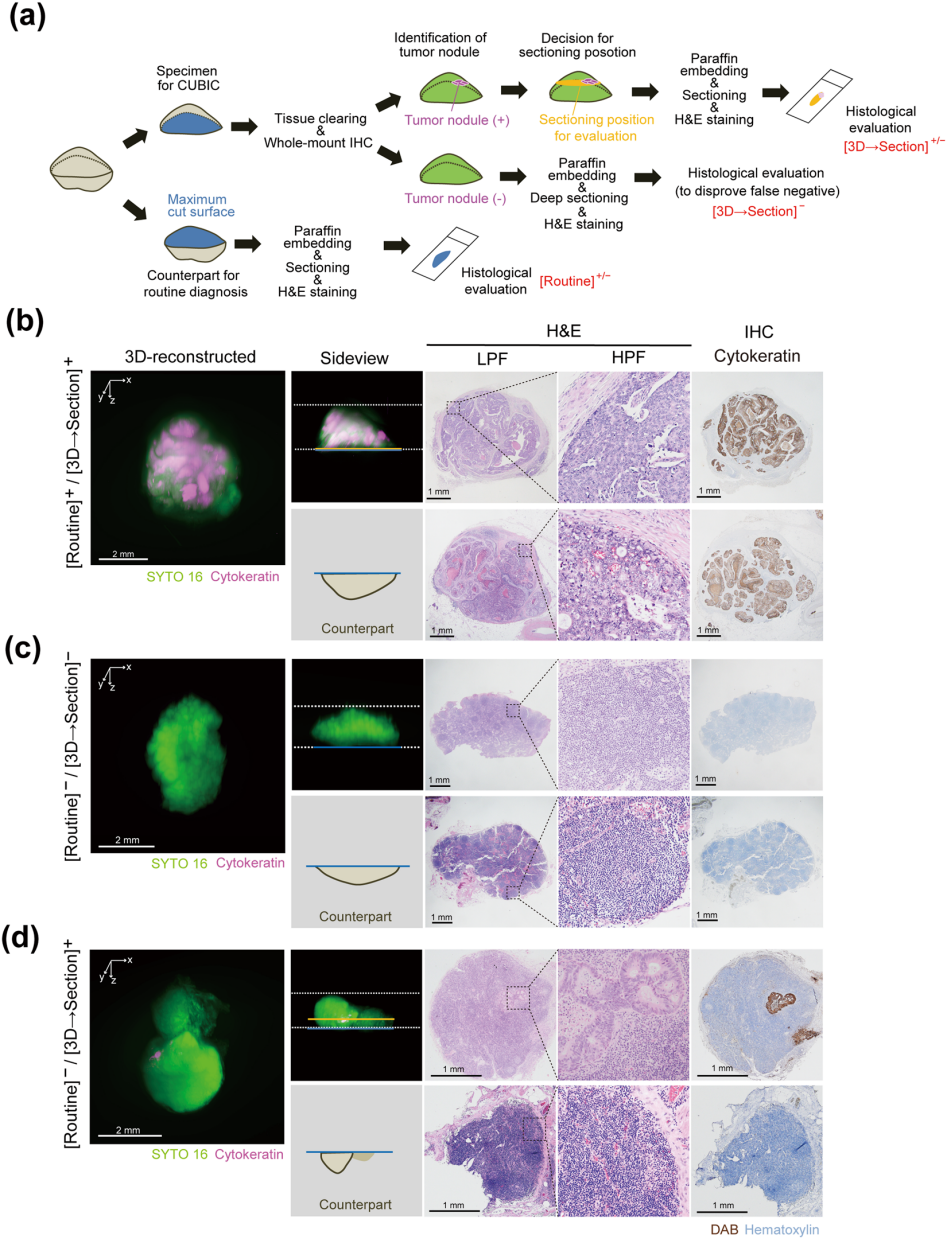
3D clinical pathology of metastatic carcinoma nodules in lymph nodes with CUBIC. (**a**) Schematic diagram of the metastasis screening protocol. Formaldehyde-fixed lymph nodes accompanying surgical specimens of colorectal cancer were cut in half. One half was subjected to the histological evaluation according to routine pathological diagnostic method in hospitals (Counterpart for routine diagnosis). The other half was subjected to CUBIC screening, which was cleared and immunostained with an Alexa Fluor 647-conjugated anti-cytokeratin antibody for LSFM imaging (z-stack: 5 μm/slice). If a suspicious tumor nodule was detected in the 3D image, the x-y plane including this nodule was sectioned for the subsequent evaluation with H&E and immunohistochemical staining. Otherwise the sample was deeply sectioned to disprove the false negative. IHC, immunohistochemistry. (**b–d**) The reconstructed 3D images for screening and H&E or immunohistochemical staining for final histological evaluation after the CUBIC screening. The 3D images were obtained with LSFM (z-stack: 5 μm/slice). (**b**) [Routine]^+^/[3D → Section]^+^ lymph node contained obvious metastatic carcinoma nodules which was positive in both routine diagnosis method and CUBIC screening. (**c**) [Routine]^−^/[3D → Section]^−^ lymph node did not contain any metastasis, confirmed both routine diagnosis and CUBIC screening. (**d**) [Routine]^−^/[3D → Section]^+^ lymph node contained small carcinoma nodules which were only identified by CUBIC screening but were not detected by routine diagnosis method.

Finally, we tested the practical effectiveness of 3D pathology on prospective clinicopathological examination. For this purpose, we performed a CUBIC-based screening to detect metastatic colorectal adenocarcinoma nodules in lymph nodes (Fig. 6a). In this screening, we used half-cut lymph nodes accompanying surgical specimens of colorectal cancer (see also the Methods section). One half of each cut lymph node was subjected to paraffin-embedding, sectioning, and H&E staining, followed by histological evaluation at the maximum cut surface, which was based on a routine pathological diagnostic method in the pathology department in hospitals. While in principle this routine method has sensitivity of practical significance, it is unable to detect minor tumor nodules which are macroscopically unrecognizable and away from the cut surface. Therefore, we hypothesised that 3D imaging after whole-mount immunostaining with a tumor-specific marker might further improve the sensitivity for the detection of metastatic carcinoma. To verify this hypothesis, the other half of the cut lymph node was cleared, and immunostained with anti-cytokeratin antibody conjugated with Alexa Fluor 647 (Figs 2a and 6a). The cleared and stained specimen was then imaged with LSFM, followed by section-based histological evaluation for a final diagnosis (Fig. 6a).

In this CUBIC-based screening, we tested surgical lymph nodes samples [total 74 lymph nodes out of 7 patients who had been diagnosed as having metastasis at least in a single lymph node (>pT1) by routine diagnosis in the hospital. The stained specimens were subjected to 3D imaging followed by 2D sectioning and H&E staining of suspicious signals (3D → Section). This two-step evaluation enabled us to (1) definitively confirm the tumor cells from our standard section-based criteria, and (2) exclude artefactual signals from autofluorescence of red blood cells or blood vessels, dust, bubbles, or antibody aggregates. Therefore, the results were classified as (1) positive in both a routine diagnosis method and a CUBIC-based screening ([Routine]^+^/[3D → Section]^+^), 2) negative in both a routine diagnosis method and a CUBIC-based screening ([Routine]^−^/[3D → Section]^−^), (3) negative in a routine diagnosis method but positive in a CUBIC-based screening ([Routine]^−^/[3D → Section]^+^), and (4) positive in a routine diagnosis method but negative in a CUBIC-based screening ([Routine]^+^/[3D → Section]^−^) (Fig. 6b–d).

In the [Routine]^+^/[3D → Section]^+^ case, the 3D imaging and the following section-based evaluation at the maximum surface clearly depicted the carcinoma nodules inside the specimen (Fig. 6b). In addition, H&E and cytokeratin immunostaining after the CUBIC procedure gave comparable results, suggesting that the CUBIC-based screening can be added on to the routine histopathological procedure. Note that such large tumor nodules remained opaque and were macroscopically visible even after CUBIC clearing (Supplementary Fig. 7), suggesting the usefulness of CUBIC for gross pathology as well.

On the other hand, some of the specimens did not include any significant signal for possible tumor nodules both by routine diagnosis or CUBIC-based screening (Fig. 6c). Therefore, these cases were classified as [Routine]^−^/[3D → Section]^−^. After 3D imaging, these negative samples were deeply sectioned for denying false negative. In the case that a potent tumor nodule was detected for the first time in 3D image, the section plane including the nodule position was then prepared and histologically evaluated (Fig. 6a). Some of the cases, in which the 3D image contained the artefactual signals as above but no tumor cell was detected in the following section-based evaluation (Supplementary Fig. 8), were reclassified as [Routine]^−^/[3D → Section]^−^. However, the remaining cases indeed contained a small tumor cell nodule inside, which was further confirmed by the section-based evaluation at the position and thus classified as [Routine]^−^/[3D → Section]^+^ (Fig. 6d). Importantly, for diagnosis of [3D → Section]^+/−^, the suspected nodular signal in the 3D image was subsequently examined by sectioning and H&E staining in all of the cases. Because of the experimental design, [3D → Section]^−^ data did not include false positive. In addition, since all tumor nodules identified in routine examination were again detected in CUBIC-based screening, there was no case of [Routine]^+^/[3D → Section]^−^.

The results are summarized in Table 1. Four lymph nodes out of 74 were newly diagnosed as positive metastasis only by the CUBIC-based screening. Because [3D → Section]^−^ samples were subjected to a deep-cut sectioning for excluding false negative, the numbers in the CUBIC-based screening can be considered as true positive values. Therefore, these results yielded the sensitivity of a routine diagnosis method as 85.2% (23/27) with four false negative. This means that CUBIC-based screening provided 14.8% (4/27) improved sensitivity over the routine method. Taken together, the 3D pathological screening thus can increase the sensitivity of this definitive diagnosis in the clinical evaluation of cancer metastasis.

**Table 1:** Performance of CUBIC-based lymph nodes metastasis screening.

		3D → sectioning	
		Positive (n = 27)	Negative (n = 47)
Routine method	Positive (n = 23)	23	0
	Negative (n = 51)	4	47

74 lymph nodes were selected from the cases in which at least one lymph node, among all regional lymph nodes for each case, was diagnosed as positive for metastasis (>pT1) by the routine diagnosis method. Four lymph nodes out of 74 were newly diagnosed as positive for metastasis only by the CUBIC-based screening (3D → sectioning), making the sensitivity of the routine diagnosis method 85.2% (23/27) with four false negative, i.e. 14.8% (4/27) improvement in sensitivity with CUBIC.

## Discussion

Tissue-clearing and 3D-imaging techniques enable comprehensive analysis of cells throughout whole organs and organisms^8, 25^. These techniques have been further applied to optical clearing, histological labelling and 3D imaging of human samples^11, 13–16, 40, 41^. In this study, we investigated the applicability of tissue-clearing and 3D-imaging techniques to histopathological diagnosis of clinical specimens. We used CUBIC for this purpose because CUBIC fulfills the criteria required for the pathological examination of clinical specimens such as clearing efficiency, ease of use, safety of the protocol, and proven performance in 3D imaging with LSFM and confocal microscopy^9, 34, 38^. We tested the current version of CUBIC clearing protocol with various human organs and found that the lung and the lymph node were efficiently cleared and could be used for the following feasibility study (Fig. 1). We further found that CUBIC could be used for clearing, staining and 3D imaging of not only newly fixed samples but also paraffin-embedded tissues stored in the pathological archives of hospitals (Figs 2–5 and Supplementary Figs 2–3). This point is practically important for the usage of other pathological archives in many hospitals. Furthermore, the human samples after CUBIC could be evaluated by a conventional pathological examination with H&E staining. These results highlighted that the tissue-clearing and 3D-imaging techniques based on CUBIC could be applied to clinicopathological examination of human samples without losing its compatibility with the conventional pathology methods.

Tissue-clearing and 3D-imaging techniques have great advantages in objective and quantitative analysis. In this study, we demonstrated these advantages in the pathological examination of clinical specimens by using CUBIC. Tissue clearing and 3D imaging of lymph nodes of malignant lymphoma identified nuclear and structural atypia, suggesting the objectivity and quantitativeness of 3D pathological examination (Fig. 5). Furthermore, 3D pathological examination could increase the sensitivity of diagnostic testing by detecting small metastatic colorectal adenocarcinoma nodules in the associated lymph nodes (Fig. 6 and Table 1). In this screening, we adopted a two-step evaluation (CUBIC-based 3D imaging and subsequent 2D histopathological evaluation using H&E-stained sections of the suspicious signals). The two-step evaluation successfully helped us to find minor metastatic lesions which could not be detected by conventional pathological examination. Although the CUBIC-based 3D evaluation in this study could not completely distinguish artefactual signals from true metastatic lesion-derived signals without the subsequent H&E staining, further technical advancements in clearing, staining and 3D imaging should realize a complete 3D imaging-based metastasis screening system. Collectively, these results support the idea that the retrospective and prospective 3D clinical pathology can improve the diagnostic capability of routine pathological examinations by increasing its objectivity, quantitativeness and sensitivity. This 3D clinical pathology would also facilitate the accumulation of histopathological knowledge based on the 3D structure of diseased organs.

Concerning the compatibility of CUBIC with immunohistochemistry, we and other researchers have tested dozens of antibodies and stains which work with the CUBIC-treated specimens^9, 12, 34, 39, 42–45^, suggesting the usefulness of CUBIC in a wide range of immunohistochemistry applications in histopathological studies and diagnosis. This point is also important because fluorescent labelling by genetic tools cannot be utilized for human specimens^8^. However, while most of the clearing methods have been shown as to be compatible with histological staining, including human tissue samples^9, 11–15, 17, 34, 43^ or more complicated multiplex staining or *in situ* hybridization for RNA detection^41, 46^, only few critical parameters for efficient penetration of stains, antibodies or probes into the 3D tissue have been identified so far. Although electrophoresis or pressure-driven 3D staining have been tested^12, 47, 48^, establishment of a simple but robust staining method based only on diffusion penetration will be needed to accelerate further applications of tissue-clearing and 3D-imaging techniques for 3D clinical pathology. Ideally, further identification of staining reagents based on small molecules such as short peptides^49^ and nucleic acids (e.g. aptamers)^50^ will also facilitate 3D clinical pathology. Antigenicity is also a general issue in immunohistochemistry. Since antigenicity is dependent on not only tissue clearing but also other histological preparation conditions (e.g. fixation), the detailed histological preparation conditions should be described for each working antibody.

The other remaining concern is the tissue clearing efficiency. In this study, we demonstrated the applicability of the first-generation CUBIC reagents to lymph nodes and lung. For other human organs, however, these CUBIC reagents need to be updated so as to improve clearing efficiency. The efficiency of tissue clearing for human tissues seemed generally worse than that for mouse tissues in our study, probably due to some significant difference of individual cell size, tightness between cells, and/or rigidity of stroma components. In particular, the difficulty on complete decolorization of endogenous chromophores inside colored human tissues is an issue to be addressed (Supplementary Fig. 1). Furthermore, melanin, a pigment derived from tyrosine via a multistep reaction, often becomes an obstacle for tissue decolorization and no efficient protocol has been reported for removal of this type of pigment^8^. Since the previous studies^18, 34, 51^ only screened a relatively small number of chemicals, more potent tissue-clearing and decolorizing chemicals should be identified by a large-scale chemical screening in the future.

In conclusion, the CUBIC-based 3D clinical pathology (CUBIC pathology) demonstrated here provides an opportunity to increase the capability of diagnostic pathology. Further development and optimization of the protocol would facilitate the future of routine pathology with 3D examination, and will allow pathologists to efficiently accumulate 3D pathological findings, leading to the establishment of a novel field of medical science.

## Methods

### The CUBIC clearing

Two CUBIC reagents were prepared as previously reported^34, 38^. For preparation of CUBIC-treated samples, the formaldehyde-fixed organs were resected into tissue blocks (2–5 mm × 2–5 mm × 0.5–3 mm), followed by wash with PBS. These tissue blocks were immersed into 8–10 ml of 50% (v/v) Sca*l*eCUBIC-1 (designated as CUBIC-1) reagent (1: 1 mixture of water: CUBIC-1) for 1 day and further immersed in 8–10 ml of CUBIC-1 reagent for 3 days (Figs 1–6) or 7 days (Supplementary Fig. 1). If counterstain was required, 1 μM SYTO 16 (Life Technologies Inc., Rockville, MD, S7578) was added into CUBIC-1 reagent on the second day. Subsequently, these samples were washed with PBS and immersed into 8–10 ml of 50% (v/v) Sca*l*eCUBIC-2 (designated as CUBIC-2) reagent (1: 1 mixture of water: CUBIC-2) for 1 day and further immersed in 8–10 ml of CUBIC-2 reagent for 2 days (Fig. 1), 1 day (Figs 2–6), or 7 days (Supplementary Fig. 1).

### Pathology specimens

Tissue specimens used in Figs 1–5 were derived from the patients who underwent pathological dissection at Osaka University Hospital from 2013 to 2015. Lymph node specimens used in Fig. 6 were derived from the patients who underwent surgery for colorectal carcinoma in Kinki Central Hospital in 2015 to 2016. In this study, we used half-cut lymph nodes from the cases in which at least one lymph node, among all regional lymph nodes belonging to each case, was diagnosed as positive for metastasis (>pT1) by the routine diagnosis method. In diagnostic practice, formaldehyde-fixed lymph nodes accompanying surgical specimens of colorectal cancer were evaluated by sectioning to check subgrossly for metastatic nodules. If a massive metastatic nodule exists in a lymph node, it is sectioned at the maximum cut surface crossing the tumor nodule. If a lymph node is small and with no macroscopically recognizable metastatic nodule, it is often sectioned at an arbitrary maximum cut surface. Such macroscopic diagnosis is carefully performed by pathologists according to the guideline^52^. Thus, we used such half-cut lymph node specimens in the screening shown in Fig. 6. The study was approved by the Ethical Review Board of the Graduate School of Medicine, Osaka University (No. 14470), Kinki Central Hospital (No. 263), and the Graduate School of Medicine, The University of Tokyo (No. 10917), and was performed in accordance with the committee guidelines and regulations. Informed consent was obtained from all patients.

### Mouse organs

Mouse organs used as control in Fig. 1 and Supplementary Fig. 1 were prepared from 12-week-old female C57BL/6 J mice. Popliteal lymph nodes were prepared from 7-week-old female C57BL/6 J mice immunized with Keyhole limpet hemocyanin (KLH) in complete Freund’s adjuvant (CFA) in the footpad. Mice were bred at the Animal Resource Center for Infectious Diseases, Research Institute for Microbial Diseases and Immunology Frontier Research Center, Osaka University. All animal procedures were approved by licensing committees of Infectious Diseases, Research Institute for Microbial Diseases and Immunology Frontier Research Center, Osaka University, and were conducted according to the institutional guidelines.

### Microscopy and Image analysis

3D images were acquired with confocal microscopy (Zeiss LSM710 Confocal/Multiphoton, Carl-Zeiss, Jena, Germany). All raw image data were exported as czi files, followed by analysis with Imaris software (version 7.7.2, Bitplane, Zurich, Switzerland). All images were obtained with constant laser power. 3D images were also acquired with light-sheet fluorescence microscopy (Ultramicroscope, LaVision BioTec, Germany) as reported previously^10, 34^. All raw image data were collected in a lossless 16-bit TIFF format. 3D-rendered images were visualized, captured and analyzed with Imaris software (version 7.7.2, Bitplane, Zurich, Switzerland).

Quantification of nuclear atypia and pleomorphism of lymphoma cells and lymphocytes was performed by using ImageJ software (Fig. 5d)^53^. 10 independent optical slices were selected from the z-stacked images of malignant lymphoma and normal lymph node shown in Fig. 5c. 10 lymphoma cells or lymphocytes per respective slices were randomly selected and contours of these cells were tracked by “Freehand selections” tool of ImageJ software, which were analyzed to evaluate their nuclear area and circularity. Lymphoma cells and lymphocytes were morphologically distinguished from other kinds of cells, especially from antigen presenting cells (APCs), because lymphoma cells and lymphocytes have scant cytoplasm whereas APCs have abundant and clear cytoplasm. For calculating nuclear area of lymphoid cells, the “Measure” function of ImageJ software was used. For calculating nuclear circularity, the “Circularity” plugin was used (https://imagej.nih.gov/ij/plugins/circularity.html). Circularity is defined as the formula: circularity = 4π(area/perimeter^2^), which gives the value = 1 when the object is a precise circle.

### Whole-mount 3D immunohistochemistry

Whole-mount immunohistochemistry protocol for CUBIC samples was done according to the previous paper^34^. Tissue samples were treated with CUBIC-1 reagent for 3 days, washed with PBS, immersed in 30% (w/v) sucrose in PBS, and frozen in O.C.T. compound at −80 °C overnight. The frozen samples were then thawed, washed with PBS, and subjected to immunostaining with the 1:50 diluted fluorescent-labeled antibodies in 0.1% (v/v) Triton X-100 in PBS for 3 days at room temperature. The stained samples were then washed with PBS several times at room temperature, followed by cross-linking in 1% PFA in PBS for 3-4 hours at room temperature. The stained samples were then immersed into 50% CUBIC-2 reagent for 1 day and CUBIC-2 reagent for 1 day. The following antibodies were used for the staining: Alexa Fluor 647-conjugated anti-α-smooth muscle actin (α-SMA) antibody (abcam, Cambridge, UK, ab196919) for human lung (Fig. 2b and Supplementary Fig. 2), and Alexa Fluor 647-conjugated anti-Pan cytokeratin antibody (C-11) (Cell signaling, Danvers, MA, #4528) for human lymph nodes (Fig. 6b–d). For actin staining, Alexa Fluor 594-labeled phalloidin (ThermoFisher Scientific, Waltham, MA, A12381) was used with the 1:25 dilution in PBS (Figs 2d and 5b).

### Whole-mount 3D staining with Congo Red and Thioflavin T

Whole-mount Congo Red and Thioflavin T staining were performed for amyloid staining according the standard protocol. For Congo Red staining, tissue samples were treated with CUBIC-1 reagent for 3 days, washed with PBS, immersed in 30% (w/v) sucrose in PBS, and frozen in O.C.T. compound at −80 °C overnight. The frozen samples were then thawed, washed with PBS, and immersed into alcoholic salt solution (saturated NaCl in 80% alcohol) at 37 °C for 2 days. These samples were then immersed into working Congo Red solution (Saturated Congo Red in 80% alcohol saturated with NaCl, in which 1/100 (v/v) of 1% NaOH solution was added just before use) at 37 °C for 2 days. The stained samples were washed consecutively with 100%, 90%, 80%, 70% ethanol diluted with water. Each washing time was within the range of 1–5 minutes, according to the degree of decolorization. Further, the samples were washed with PBS for 30 minutes, and immersed into 50% CUBIC-2 reagent for 1 day and CUBIC-2 reagent for 1 day. Fluorescence signal of Congo Red was measured by excitation at both 561 nm and 594 nm (confocal microscopy) or at 594 nm (LSFM). For Thioflavin T staining, tissue samples were treated with CUBIC-1 reagent for 3 days, washed with PBS, immersed in 30% (w/v) sucrose in PBS, and frozen in O.C.T. compound at −80 °C overnight. The frozen samples were then thawed, washed with PBS, and immersed into working Thioflavin T solution (50 mM Thioflavin T in PBS) at room temperature for 3 days. The samples were washed with PBS for 6 hours, and immersed into 50% CUBIC-2 reagent for 1 day and CUBIC-2 reagent for 1 day. Fluorescence signal of Thioflavin T was measured by excitation at 405 nm.

### Deparaffinization of tissue from paraffin-embedded tissue blocks

Deparaffinization of tissues from paraffin-embedded tissue blocks was performed according to the protocols shown in Figs 4a and 5a. Tissues were cut out with a blade (Fig. 4) or punched out by a φ 4-mm biopsy punch (Fig. 5 and Supplementary Fig. 6) from paraffin-embedded tissue blocks, followed by heating at 65 °C on heatblock until paraffin was macroscopically removed from tissues. These samples were washed with Hemo-De (FALMA, CS-1001) overnight at room temperature, followed by further wash with Hemo-De for 3 hours. The samples were washed with 100%, 90%, 80%, 70% ethanol diluted with water for 3 hours respectively, which was further washed with PBS for 6 hours, before being subjected to CUBIC tissue-clearing protocol.

### Statistical analyses

Statistical analyses were performed using Microsoft Excel or EZR version 3.3.1(2016–06–21)^54^. The normality of the two sets of data was evaluated by Kolmogorov-Smirnov test at the significance level of 0.05. When the normality was confirmed in the groups, the homogeneity of variance was tested by F-test at the significance level of 0.05. When two groups were normally distributed with or without equal variance, Student’s t-test or Welch’s t-test were applied, respectively. In this study, Welch’s t-test was used in Fig. 5d.

## Electronic supplementary material

Supplementary information

Supplementary Movie 1

Supplementary Movie 2

Supplementary Movie 3

Supplementary Movie 4

## References

[1] Marcum RG, Wellings SR (1969). Subgross pathology of the human breast: method and initial observations. J. Natl. Cancer Inst..

[2] Wellings SR, Jensen HM (1973). On the origin and progression of ductal carcinoma in the human breast. J. Natl. Cancer Inst..

[3] Wellings SR, Jensen HM, Marcum RG (1975). An atlas of subgross pathology of the human breast with special reference to possible precancerous lesions. J. Natl. Cancer Inst..

[4] Marchio C, Sapino A, Arisio R, Bussolati G (2006). A new vision of tubular and tubulo-lobular carcinomas of the breast, as revealed by 3-D modelling. Histopathology.

[5] Sun L, Wang D, Zubovits JT, Yaffe MJ, Clarke GM (2009). An improved processing method for breast whole-mount serial sections for three-dimensional histopathology imaging. Am. J. Clin. Pathol..

[6] Norton KA (2012). Automated reconstruction algorithm for identification of 3D architectures of cribriform ductal carcinoma *in situ*. PLoS One.

[7] Booth ME (2015). Three-dimensional reconstruction of ductal carcinoma *in situ* with virtual slides. Histopathology.

[8] Susaki EA, Ueda HR (2016). Whole-body and Whole-Organ Clearing and Imaging Techniques with Single-Cell Resolution: Toward Organism-Level Systems Biology in Mammals. Cell Chem. Biol..

[9] Tainaka K (2014). Whole-body imaging with single-cell resolution by tissue decolorization. Cell.

[10] Dodt HU (2007). Ultramicroscopy: Three-dimensional visualization of neuronal networks in the whole mouse brain. Nat. Methods.

[11] Hama H (2015). Sca*l*eS: an optical clearing palette for biological imaging. Nat. Neurosci..

[12] Lee E (2016). ACT-PRESTO: Rapid and consistent tissue clearing and labeling method for 3-dimensional (3D) imaging. Sci. Rep..

[13] Pan C (2016). Shrinkage-mediated imaging of entire organs and organisms using uDISCO. Nat. Methods.

[14] Chung K (2013). Structural and molecular interrogation of intact biological systems. Nature.

[15] Yang B (2014). Single-cell phenotyping within transparent intact tissue through whole-body clearing. Cell.

[16] Liebmann T (2016). Three-Dimensional Study of Alzheimer’s Disease Hallmarks Using the iDISCO Clearing Method. Cell Rep..

[17] Renier N (2014). iDISCO: a simple, rapid method to immunolabel large tissue samples for volume imaging. Cell.

[18] Becker K, Jährling N, Saghafi S, Weiler R, Dodt HU (2012). Chemical clearing and dehydration of GFP expressing mouse brains. PLoS One.

[19] Ertürk A (2012). Three-dimensional imaging of solvent-cleared organs using 3DISCO. Nat. Protoc..

[20] Hama H (2011). Sca*l*e: a chemical approach for fluorescence imaging and reconstruction of transparent mouse brain. Nat. Neurosci..

[21] Ke MT, Fujimoto S, Imai T (2013). SeeDB: a simple and morphology-preserving optical clearing agent for neuronal circuit reconstruction. Nat. Neurosci..

[22] Kuwajima T (2013). ClearT: a detergent- and solvent-free clearing method for neuronal and non-neuronal tissue. Development.

[23] Aoyagi Y, Kawakami R, Osanai H, Hibi T, Nemoto T (2015). A rapid optical clearing protocol using 2,2′-thiodiethanol for microscopic observation of fixed mouse brain. PLoS One.

[24] Costantini I (2015). A versatile clearing agent for multi-modal brain imaging. Sci. Rep..

[25] Tainaka K, Kuno A, Kubota SI, Murakami T, Ueda HR (2016). Chemical Principles in Tissue Clearing and Staining Protocols for Whole-Body Cell Profiling. Annu. Rev. Cell Dev. Biol..

[26] Olson E, Levene MJ, Torres R (2016). Multiphoton microscopy with clearing for three dimensional histology of kidney biopsies. Biomed. Opt. Express.

[27] Torres RVS, Levene MJ (2014). High-resolution, 2- and 3-dimensional imaging of uncut, unembedded tissue biopsy samples. Arch. Pathol. Lab. Med..

[28] Sommer G (2015). Biomechanical properties and microstructure of human ventricular myocardium. Acta. Biomater..

[29] Lai HM (2016). Rationalisation and Validation of an Acrylamide-Free Procedure in Three-Dimensional Histological Imaging. PLoS One.

[30] Liu AK (2016). Bringing CLARITY to the human brain: visualization of Lewy pathology in three dimensions. Neuropathol. Appl. Neurobiol..

[31] Murata, T. *et al*. Three-dimensional evaluation of subclinical extension of extramammary Paget’s disease: Visualization of histological border and its comparison to clinical border. *Br. J. Dermatol*. (2016). 10.1111/bjd.15282 28028810

[32] Neckel PH, Mattheus U, Hirt B, Just L, Mack AF (2016). Large-scale tissue clearing (PACT): Technical evaluation and new perspectives in immunofluorescence, histology, and ultrastructure. Sci. Rep..

[33] van Royen ME (2016). Three-dimensional microscopic analysis of clinical prostate specimens. Histopathology.

[34] Susaki EA (2014). Whole-brain imaging with single-cell resolution using chemical cocktails and computational analysis. Cell.

[35] Fumoto S, Nishimura K, Nishida K, Kawakami S (2016). Three-Dimensional Imaging of the Intracellular Fate of Plasmid DNA and Transgene Expression: ZsGreen1 and Tissue Clearing Method CUBIC Are an Optimal Combination for Multicolor Deep Imaging in Murine Tissues. PLoS One.

[36] Nehrhoff I (2016). 3D imaging in CUBIC-cleared mouse heart tissue: going deeper. Biomed. Opt. Express.

[37] Stefaniuk M (2016). Light-sheet microscopy imaging of a whole cleared rat brain with Thy1-GFP transgene. Sci. Rep..

[38] Susaki EA (2015). Advanced CUBIC protocols for whole-brain and whole-body clearing and imaging. Nat. Protoc..

[39] Lloyd-Lewis B (2016). Imaging the mammary gland and mammary tumours in 3D: optical tissue clearing and immunofluorescence methods. Breast Cancer Res..

[40] Casoni F (2016). Development of the neurons controlling fertility in humans: new insights from 3D imaging and transparent fetal brains. Development.

[41] Murray E (2015). Simple, Scalable Proteomic Imaging for High-Dimensional Profiling of Intact Systems. Cell.

[42] Davis FM (2016). Single-cell lineage tracing in the mammary gland reveals stochastic clonal dispersion of stem/progenitor cell progeny. Nat. Commun..

[43] Hirashima T, Adachi T (2015). Procedures for the quantification of whole-tissue immunofluorescence images obtained at single-cell resolution during murine tubular organ development. PLoS One.

[44] Ieyasu A (2017). An All-Recombinant Protein-Based Culture System Specifically Identifies Hematopoietic Stem Cell Maintenance Factors. Stem Cell Reports.

[45] Romanov RA (2017). Molecular interrogation of hypothalamic organization reveals distinct dopamine neuronal subtypes. Nat. Neurosci..

[46] Sylwestrak EL, Rajasethupathy P, Wright MA, Jaffe A, Deisseroth K (2016). Multiplexed Intact-Tissue Transcriptional Analysis at Cellular Resolution. Cell.

[47] Li J, Czajkowsky DM, Li X, Shao Z (2015). Fast immuno-labeling by electrophoretically driven infiltration for intact tissue imaging. Sci. Rep..

[48] Kim SY (2015). Stochastic electrotransport selectively enhances the transport of highly electromobile molecules. Proc. Natl. Acad. Sci. U. S. A..

[49] Lee S, Xie J, Chen X (2010). Peptide-based probes for targeted molecular imaging. Biochemistry.

[50] Juskowiak B (2011). Nucleic acid-based fluorescent probes and their analytical potential. Anal. Bioanal. Chem..

[51] Kurihara D, Mizuta Y, Sato Y, Higashiyama T (2015). ClearSee: a rapid optical clearing reagent for whole-plant fluorescence imaging. Development.

[52] Lanza G, Messerini L, Gafà R, Risio M (2011). Colorectal tumors: The histology report. Digestive and Liver Disease.

[53] Schneider CA, Rasband WS, Eliceiri KW (2012). NIH Image to ImageJ: 25 years of image analysis. Nat. Methods.

[54] Kanda Y (2013). Investigation of the freely available easy-to-use software ‘EZR’ for medical statistics. Bone Marrow Transplant..

