# Supplementary material for: CUBIC pathology: three-dimensional imaging for pathological diagnosis
Source: Sci Rep. 2017 Aug 24;7:9269. doi: 10.1038/s41598-017-09117-0 (PMC5571108; doi:10.1038/s41598-017-09117-0)
Supplement: Supplementary file 1 — Supplementary information [file 41598_2017_9117_MOESM1_ESM.pdf]

Supplementary Information

CUBIC pathology: three-dimensional imaging for pathological diagnosis.

Satoshi Nojima, Etsuo A. Susaki, Kyotaro Yoshida, Hiroyoshi Takemoto, Naoto Tsujimura, Shohei Iijima, Ko Takachi, Yujiro Nakahara, Shinichiro Tahara, Kenji Ohshima, Masako Kurashige, Yumiko Hori, Naoki Wada, Jun-ichiro Ikeda, Atsushi Kumanogoh, Eiichi Morii, Hiroki R. Ueda

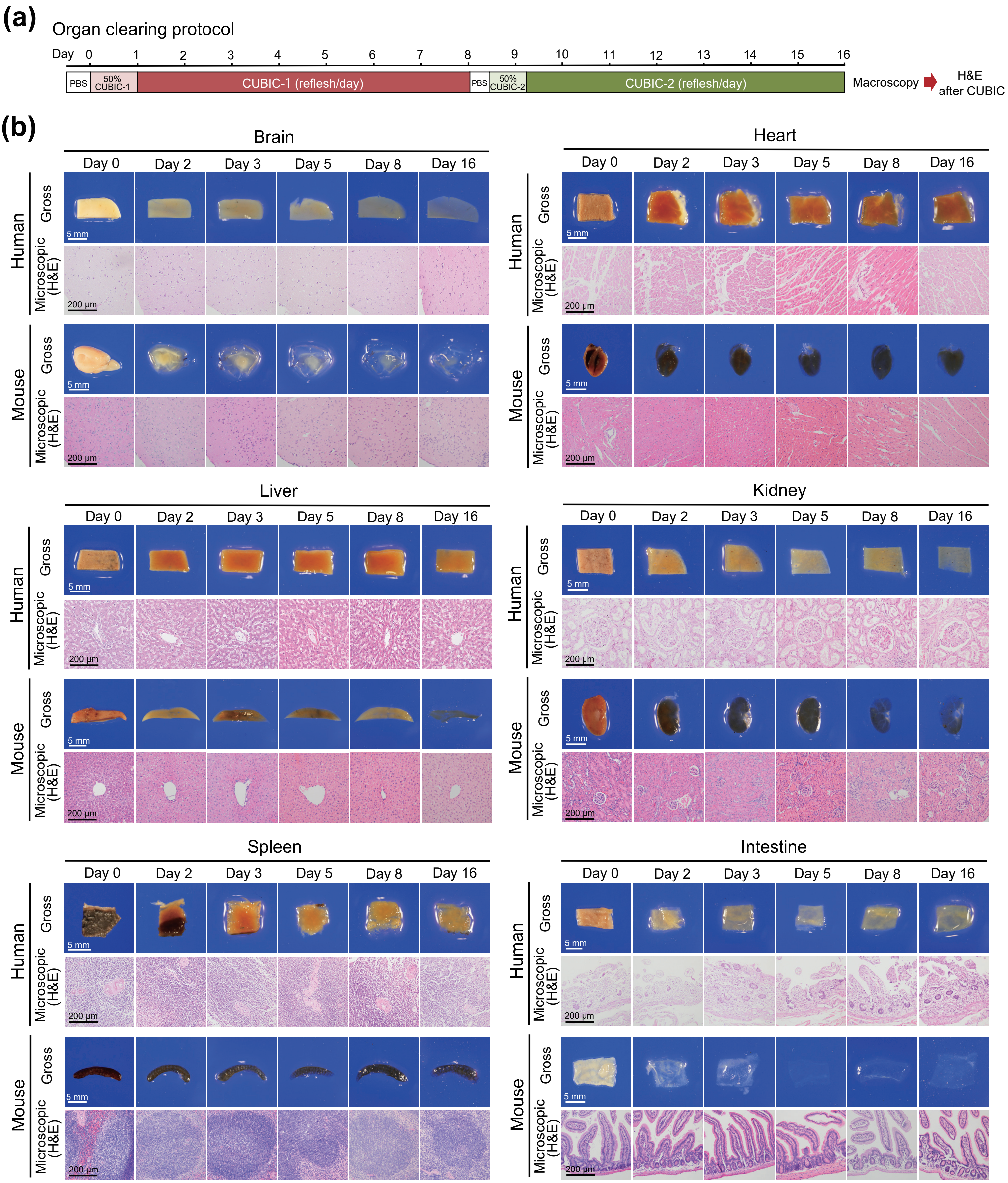

Figure S1 | Tissue clearing of various human organs with CUBIC. (a) Schematic diagram of clearing protocol for human organs. **(b)** Gross and microscopic images of human or mouse organs. After gross image acquisition at the indicated time points, tissues were washed with PBS, followed by paraffin embedding, sectioning, and H&E staining.

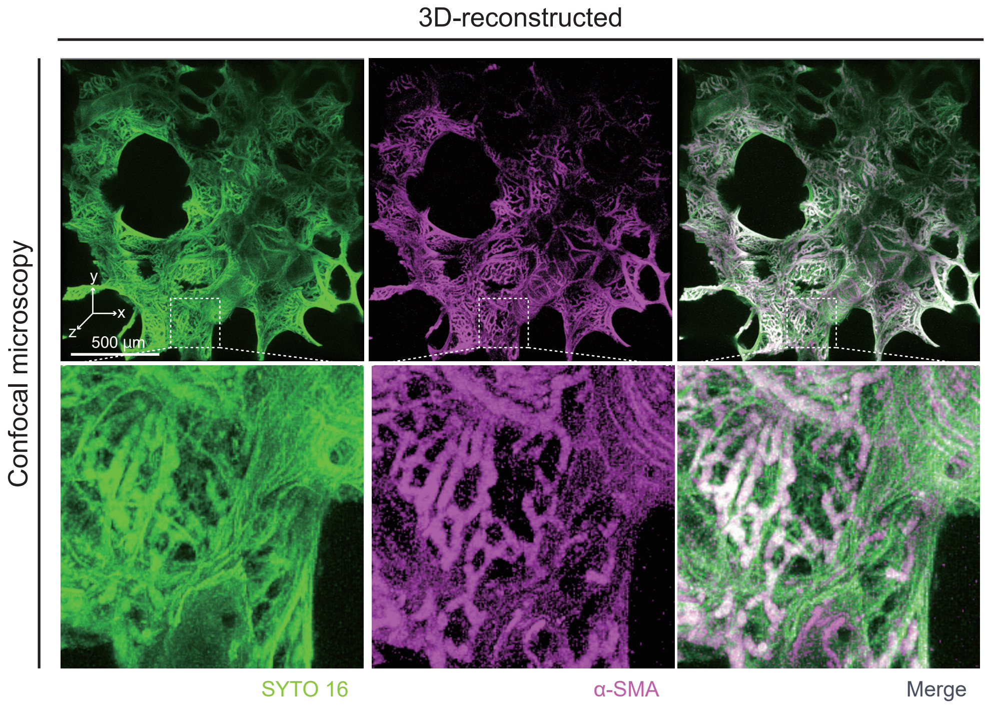

Figure S2 | 3D observation of a larger area of human lung with CUBIC. (Upper panel) 3 × 3 tiling scan and Z-stack imaging by the confocal microscopy allows the acquisition of a larger area of cleared and stained human lung. The reconstructed 3D images of the human lung tissue block stained with SYTO 16 and Alexa Fluor 647-conjugated anti-α-SMA antibody. Images were obtained by 3 × 3 tiling scan function of the confocal microscopy (z-stack: 1 µm/slice). (Lower panel) a detailed view from the 3 × 3 tile scan images, detecting sinusoidal vascular structures inside the organ.

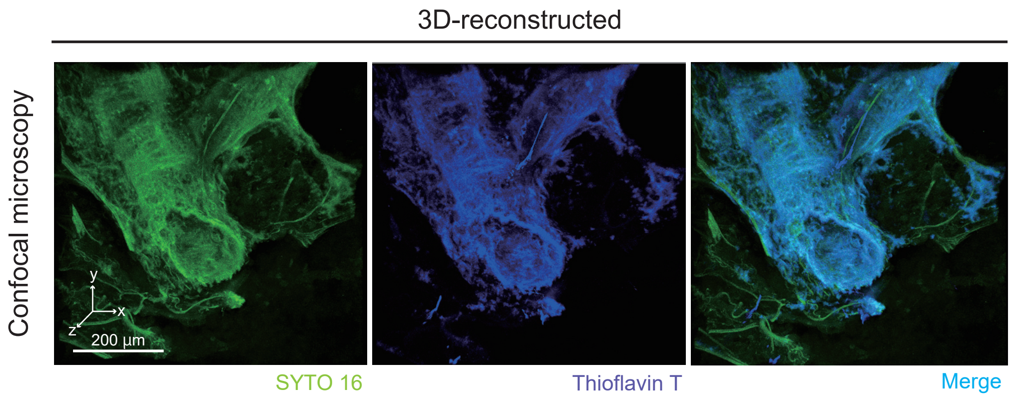

Figure S3 | Thioflavin T staining and 3D imaging of human amyloidosis lung with CUBIC. The reconstructed 3D images of the human patient's lung tissue block stained with STYO 16 and Thioflavin T, also showing that the amyloid is deposited in artery wall in the lung. The images were obtained by confocal microscopy.

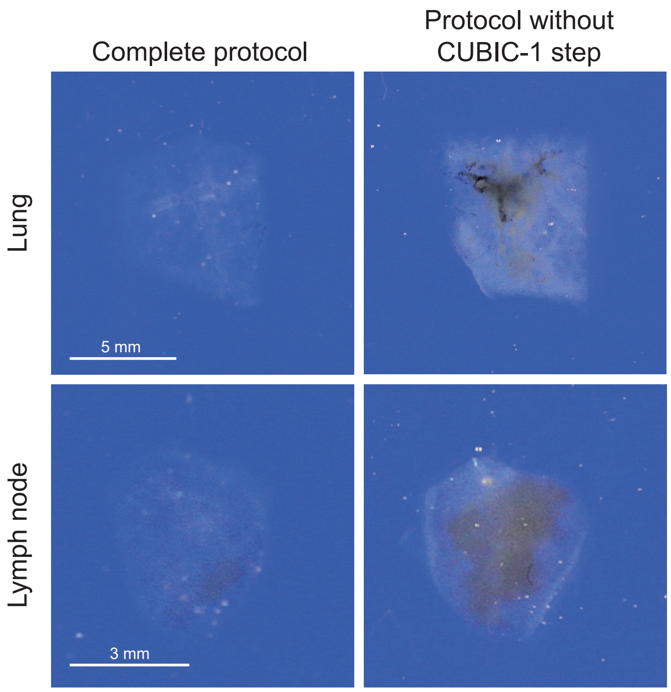

Figure S4 | The protocol without CUBIC-1 step cannot sufficiently clear deparaffinized tissue specimens. Formaldehyde-fixed human lung and lymph node tissues were embedded into paraffin-embedded tissue blocks, and then the tissues were recovered according to the protocol shown in Figure 4. The recovered tissues were then subjected to tissue-clearing procedure according to the complete protocol (with both CUBIC-1 and CUBIC-2 clearing steps) or the protocol without CUBIC-1 step (with only CUBIC-2 clearing step). Tissues processed by the protocol without the CUBIC-1 step were soaked into PBS, instead.

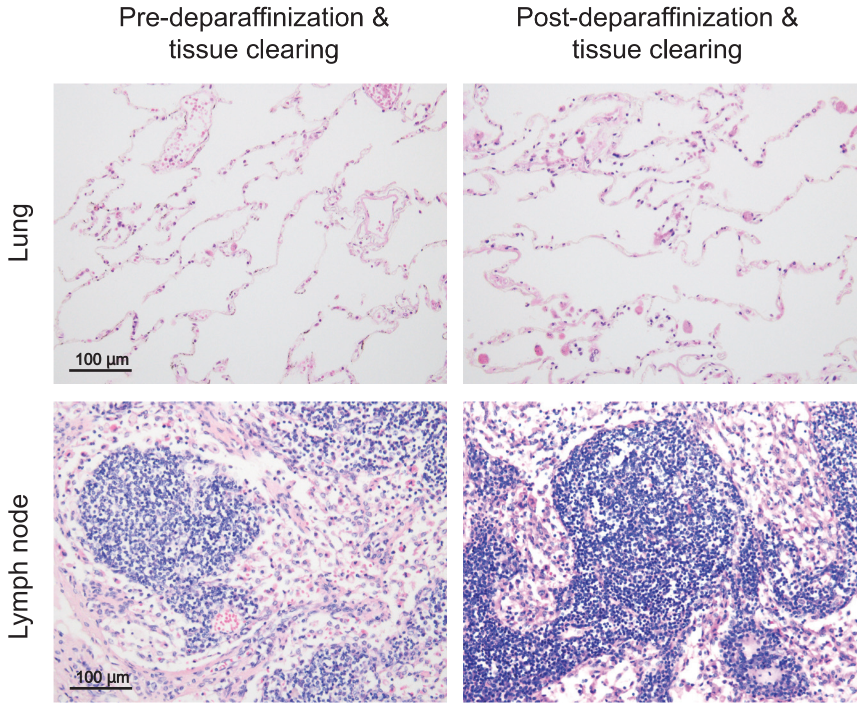

Figure S5 | The deparaffinization and tissue-clearing steps do not cause significant tissue degeneration. Formaldehyde-fixed human lung and lymph node tissues were embedded into paraffin blocks. H&E sections were made from these blocks prior to the recovery step (“Pre-deparaffinization & tissue clearing”), which were subjected to deparaffinization and tissue-clearing procedures according to a protocol similar to that in Figure 4. These specimens were re-embedded into paraffin blocks and H&E sections were generated from them (“Post-deparaffinization & tissue clearing”).

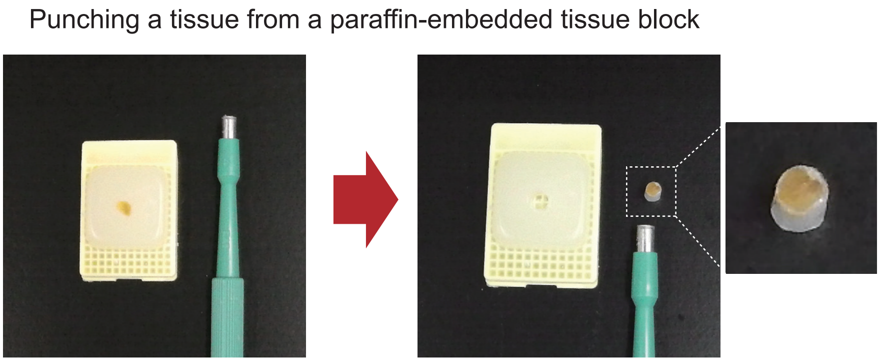

Figure S6 | A punching method for tissue clearing and 3D imaging of paraffin-embedded tissues in pathology archives with CUBIC. For preparing samples in Figure 5, the tissues were punched out from the paraffin-embedded tissue blocks by φ4 mm biopsy punch and then used for deparaffinization and tissue clearing according to the protocol shown in Figure 5a.

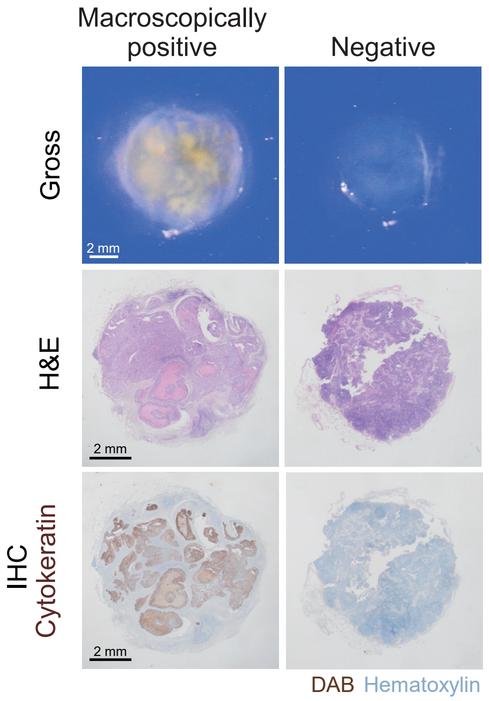

Figure S7 | Macroscopically visualized metastatic carcinoma nodules in the lymph node with CUBIC. (Upper panels) Gross images of the lymph nodes which were macroscopically positive or negative for metastatic carcinoma, related to Figures 6b and 6c. As shown in the positive case, the nodules of metastatic carcinoma showed resistance to clearing and thus they were macroscopically observable as yellowish-white masses which was distinguishable from transparent normal lymph node areas. (Middle and lower panels) Post-CUBIC evaluation by H&E and immunohistochemical staining of the samples above. After the gross image acquisition, the lymph nodes were washed with PBS, and then subsequent paraffin embedding, sectioning, H&E staining and immunohistochemistry with anti-cytokeratin antibody were performed.

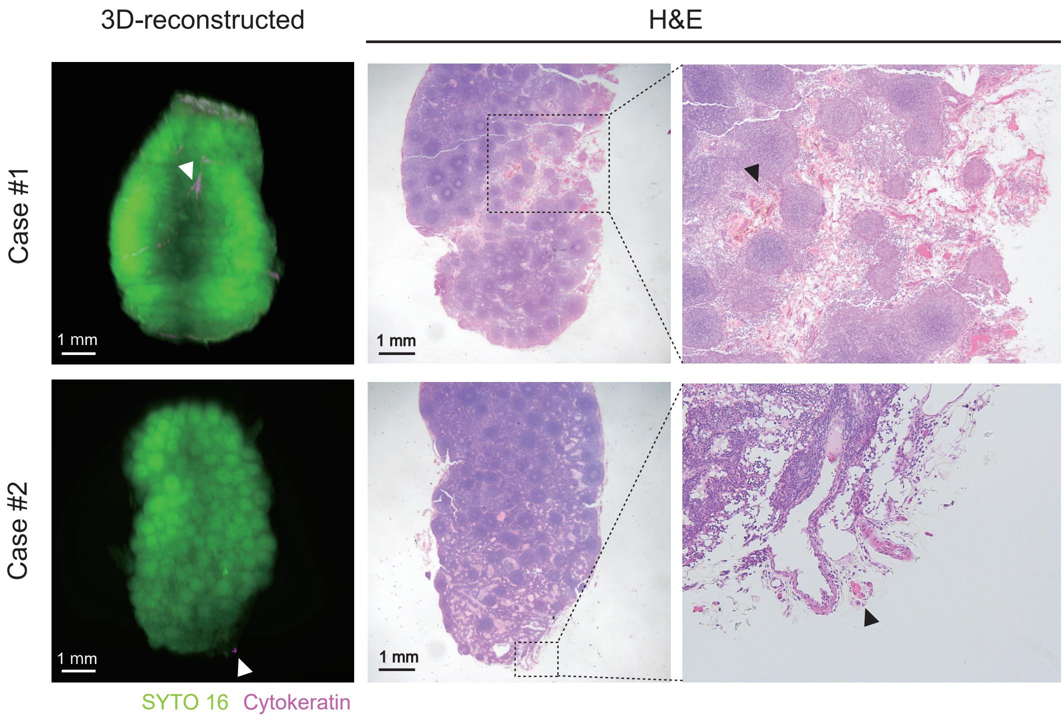

Figure S8 | 3D models of lymph nodes with artefactual signals. The reconstructed 3D images of lymph nodes in which suspicious signals were observed in 3D evaluation but these were proven to be artefact in subsequent 2D evaluation using H&E-stained sections at the suspicious signals. In the cases shown here, these signals (white arrowhead) seem to be derived from autofluorescence of small vessels with congestion and/or hemorrhage around them (black arrowhead).

SUPPLEMENTARY MOVIE LEGENDS

Supplementary Movie 1. The human lung tissue stained with an anti-α-SMA antibody and SYTO 16, related to Figure 2b.

The reconstructed 3D image of CUBIC-cleared human lung tissue stained with Alexa Fluor 647-conjugated anti-α-SMA antibody and SYTO 16, which was obtained by confocal microscopy.

Supplementary Movie 2. The SYTO 16-stained human lung tissue, related to Figure 2d.

The reconstructed 3D image of CUBIC-cleared human lung tissue stained with SYTO 16, which was obtained by LSFM.

Supplementary Movie 3. The SYTO 16-stained human lymph node tissue, related to Figure 2d.

The reconstructed 3D image of CUBIC-cleared human lymph node tissue stained with SYTO 16, which was obtained by LSFM.

Supplementary Movie 4. The Congo Red-stained human lung tissue, related to Figure 3d.

The reconstructed 3D image of CUBIC-cleared human lung tissue after Congo Red staining, which was obtained by confocal microscopy.
